# Vaccine Candidate Brucella melitensis 16M*ΔvjbR* Is Safe in a Pregnant Sheep Model and Confers Protection

**DOI:** 10.1128/mSphere.00120-20

**Published:** 2020-05-13

**Authors:** Martha E. Hensel, Daniel G. Garcia-Gonzalez, Sankar P. Chaki, Airn Hartwig, Paul W. Gordy, Richard Bowen, Thomas A. Ficht, Angela M. Arenas-Gamboa

**Affiliations:** a College of Veterinary Medicine and Biomedical Sciences, Texas A&M University, College Station, Texas, USA; b Department of Biomedical Sciences, Colorado State University, Fort Collins, Colorado, USA; University of Florida

**Keywords:** *Brucella melitensis*, brucellosis, ovine, pregnancy, veterinary vaccine development

## Abstract

As a natural host species for Brucella melitensis, pregnant sheep offer an ideal model to evaluate vaccine candidates for safety. B. melitensis strain Rev. 1 has been used almost exclusively to prevent brucellosis in small ruminants, but it causes abortions when given to pregnant animals. To evaluate the comparative safety of the candidate Brucella melitensis 16M*ΔvjbR*, pregnant sheep (*n* = 6) were vaccinated subcutaneously with 1 × 10^10^ CFU/ml of 16M*ΔvjbR* or 1 × 10^9^ CFU/ml Rev. 1 at a highly susceptible stage of gestation (approximately 70 days). 16M*ΔvjbR* resulted in only 1 abortion (1 of 6) compared with 4 of 6 (66.7%) abortions in the Rev. 1 cohort. The placenta was evaluated by culture to determine if vaccination resulted in colonization. As another measure of safety, effects of B. melitensis on the fetus/offspring (vertical transmission) was evaluated by culture and histopathology of fetal tissues to determine if vaccination prevented infection of the fetus. Vaccination with 16M*ΔvjbR* resulted in less vertical transmission than Rev. 1. To determine if vaccination was efficacious and could reduce tissue colonization in sheep, the same cohort of sheep were challenged 5 weeks postpartum by conjunctival inoculation with 1 × 10^7^ CFU/ml B. melitensis. Protection was similar between Rev. 1 and 16M*ΔvjbR*, with no statistical difference in colonization in the target organs. Overall, the 16M*ΔvjbR* vaccine was considered safer than Rev. 1 based on a reduced number of abortions and limited infection in the offspring. Future experiments are needed to further refine the vaccine dose to increase the safety margin and to evaluate protection in pregnant ewes.

**IMPORTANCE** Brucellosis is one of the most commonly reported zoonotic disease with a worldwide distribution. Of the 12 *Brucella* species, Brucella melitensis is considered the most virulent and causes reproductive failure (abortions/stillbirths) in small ruminants, which can spread the disease to other animals or to humans. Vaccination of small ruminants is a key measure used to protect both human and animal health. However, the commercially available live-attenuated vaccine for Brucella melitensis Rev. 1 retains virulence and can cause disease in animals and humans. In order to evaluate the safety and efficacy in sheep, we vaccinated pregnant sheep with 16M*ΔvjbR*. Our results indicate that 16M*ΔvjbR* was safer for use during pregnancy, provided a similar level of protection as Rev. 1, and could be considered an improved candidate for future vaccine trials.

## Abstract (precis)

Brucellosis is one of the most commonly reported zoonotic disease with a worldwide distribution. Of the 12 *Brucella* species, Brucella melitensis is considered the most virulent and causes reproductive failure (abortions/stillbirths) in small ruminants, which can spread the disease to other animals or to humans. Vaccination of small ruminants is a key measure used to protect both human and animal health. However, the commercially available live-attenuated vaccine for Brucella melitensis Rev. 1 retains virulence and can cause disease in animals and humans. In order to evaluate the safety and efficacy in sheep, we vaccinated pregnant sheep with 16M*ΔvjbR*. Our results indicate that 16M*ΔvjbR* was safer for use during pregnancy, provided a similar level of protection as Rev. 1, and could be considered an improved candidate for future vaccine trials.

## INTRODUCTION

Brucellosis is caused by a zoonotic Gram-negative, facultative intracellular bacterium, and of the 12 recognized species, Brucella melitensis is the most virulent to humans (1, 2). Sheep and goats are the natural hosts for B. melitensis, and the disease manifests as spontaneous mid- to late-term abortions with infertility and retained placenta (2). Humans become infected by ingestion of unpasteurized milk or milk products, by infectious aerosols, or through direct contact with infected animals (2). In humans, the disease is characterized by nonspecific flu-like symptoms of fever, malaise, anorexia, and joint pain (2). Since a vaccine for use in humans is not available, vaccination of animals is an important means of preventing disease in humans (3).

Live attenuated vaccines (LAVs) have been used almost exclusively to prevent brucellosis in animals because they have been proven to be the most efficacious vaccine type compared with others, such as cell extracts or DNA vaccines (4, 5). The currently approved vaccine for use in small ruminants, Brucella melitensis strain Rev. 1, is a live-attenuated mutant that has been extensively used worldwide since its identification (4). A drawback to the use of Rev. 1 during pregnancy is a variable but significant abortion rate of 40% to 80%, which can propagate disease in the flock and poses a risk for humans handling the aborted placentas/fetuses (6–8). Several attempts have been made to improve the safety of Rev. 1, such as reducing the dose or vaccination via the conjunctival mucosa, but the risk for spontaneous abortion remains (8). In addition, Rev. 1 causes a disease syndrome in humans that is similar to infection with the wild type, so it poses a risk for those administering the vaccine (9).

The 16M*ΔvjbR* vaccine was developed as a single mutant live-attenuated vaccine candidate and has been evaluated in BALB/c mice as well as immunodeficient mouse models to determine safety and efficacy. These studies found that 16M*ΔvjbR* resulted in less inflammation and persistence than strain S19, while also protecting against challenge with wild-type *Brucella* spp. (10, 11). This study expands upon previous studies in a nonpregnant mouse model, which demonstrated that 16M*ΔvjbR* stimulates a protective immune response (10–12). However, given the differences in target cell specificity, studies in the mouse model alone are insufficient to determine whether the vaccine will behave safely and efficaciously in the natural host. In light of the information gained from mouse models, the next step was to determine the safety of the vaccine candidate 16M*ΔvjbR* in a host that recapitulates natural infection events. Pregnant ewes, as a natural host, provide all of the appropriate tissue targets to fully evaluate the safety of the vaccine candidate during pregnancy as well as to determine whether the vaccine is capable of inducing protection against infection in nonpregnant animals.

## RESULTS and DISCUSSION

The currently available vaccine for small ruminants, Rev. 1, can cause abortion in pregnant animals and disease in humans (8, 9). Therefore, an improved vaccine is needed to confer protection while failing to induce adverse events, such as abortion and vaccine shedding from animals. As a natural host and strategic target for vaccination, pregnant ewes were used to determine the safety of vaccine candidate 16M*ΔvjbR* compared with Rev. 1. Pregnancy was confirmed at 60 days of gestation by ultrasonography, and ewes were then vaccinated 10 days later (day 70). Previous studies have shown that pregnant animals are most susceptible to adverse pregnancy events like abortion during midgestation (approximately 60 to 120 days of gestation) if they are exposed to wild-type B. melitensis or vaccinated with Rev. 1 during this time period (3, 8). Therefore, by vaccinating animals at approximately 70 days of gestation, we tried to replicate this period of increased susceptibility to abortion in pregnant ewes. The current vaccination strategy of whole-flock immunization means that pregnant animals have the potential to be vaccinated during vulnerable stages of pregnancy. Since Rev. 1 is only safe to use in young animals, an improved vaccine is critically needed that can be used for whole-flock vaccination campaigns without resulting in adverse pregnancy events that can lead to exposure of other sheep and humans.

### Temperature.

**FIG 1:**
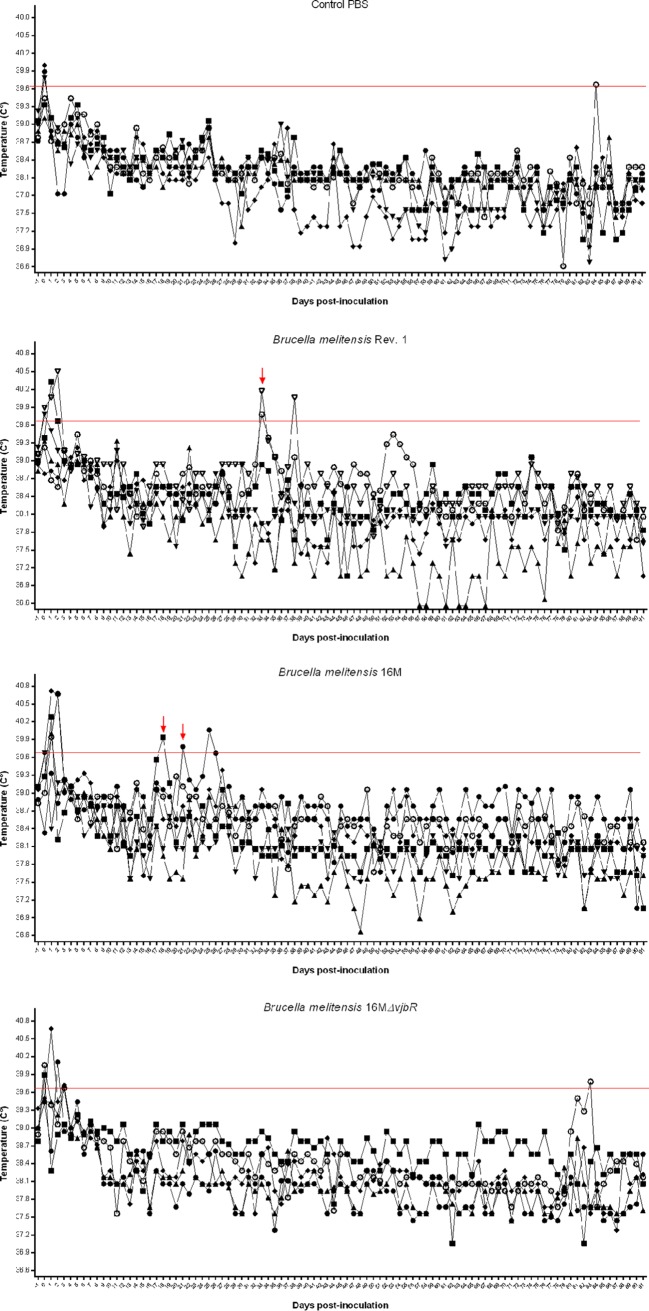
Body temperature in response to vaccination or infection. Implantable subcutaneous transponders (LifeChip) were placed in the right axillary subcutaneous space and were monitored once daily using a DAS-7000 reader (BioMedic Data Systems) for the duration of the study. Control ewes (A) were sham vaccinated with 1 ml of sterile PBS. Vaccination with 16M*ΔvjbrR* (D) was not associated with development of fever. Two pregnant sheep in the Rev. 1 (B) and 16M (C) groups each developed transient fevers (red arrows) on the day abortion occurred. Body temperatures of ≥39.7°C (red line) indicate a fever response. Temperatures are reported in degree Celsius.

Spontaneous abortion is often the first indication of brucellosis in a flock, but scant evidence is available to determine whether body temperature can be used in the small ruminant to predict infection or abortion potential (13). In order to evaluate the temperature response to the vaccines as well as wild-type (16M) B. melitensis, implantable microchips were used to measure body temperature throughout the study period. The threshold for fever was established at a temperature of ≥39.7°C (14). Similar to a previous study in goats, a transient increase in temperature was noted immediately following vaccination in all groups, which resolved by 48 h (Fig. 1A to D) (14). Since 24 h postinoculation is insufficient to establish a systemic infection, this was considered to be a stress response to handling during vaccination. None of the animals in the phosphate-buffered saline (PBS) or 16M*ΔvjbR* groups developed fever (Fig. 1D) after the initial 48-h period. Interestingly, 2 of 6 animals in the 16M and 2 of 6 ewes in the Rev. 1 groups developed transient fevers at approximately 19 to 25 days and 34 to 39 days postinoculation, respectively, during the approximate time of abortions in these groups (Fig. 1B and C). A few studies have evaluated temperature following challenge with the wild type or vaccination with Rev. 1 in sheep and goats; however, these studies did not measure temperature for the whole study period and did not note fever in response to infection or vaccination (7, 14, 15). The remaining animals in the 16M and Rev. 1 groups did not experience fever despite having spontaneous abortions, and thus, in contrast to infection in humans, fever is an unreliable indicator of systemic infection.

### Abortion.

In order to determine if we accurately modeled the events that occur following infection with 16M and vaccination with Rev. 1 during pregnancy, the ewes were evaluated twice daily for adverse pregnancy events. As expected, 100% of the ewes administered sterile PBS had normal live births, and 100% of the ewes inoculated with B. melitensis 16M aborted between 21 to 56 days postinoculation (Table 1) at the anticipated time of approximately 3 to 7 weeks postinfection (16). Additionally, 4 of 6 (67%) ewes vaccinated with Rev. 1 aborted between 18 to 44 days postinoculation (Table 1). An abortion rate of 40% to 80% is an established side effect of Rev. 1, even when used at half (5 × 10^8^ CFU/ml) or reduced doses (1 × 10^6^ CFU/ml) (6, 8). Interestingly, only 1 of 6 (16.7%) ewes from the 16M*ΔvjbR* group aborted at 40 days postinoculation (Table 1). These data indicate that 16M*ΔvjbR* is significantly attenuated compared with Rev. 1 because, even when given at a dose 10 times higher, 16M*ΔvjbR* proved safer to use during pregnancy based on a reduced number of spontaneous abortions. It remains to be evaluated if decreasing the dose would increase the margin of safety to achieve fewer abortions.

**TABLE 1:** Abortion outcomes by treatment group^*a*^

Treatment group	Day(s) postvaccination	No. (%) of abortions	No. of offspring
16MΔ*vjbR*	40	1/6 (16.7)	7
OviRev (Rev. 1)	18, 22, 23, 44	4/6 (66.7)	11
*B. melitensis* 16M	21, 22, 24, 30, 33, 56	6/6 (100)	9
PBS	0	0/6 (0)	9

a At day 70 of gestation, pregnant sheep (*n* = 6) were inoculated via subcutaneous injection with 1 × 10^10^ CFU/ml 16M*ΔvjbrR*, 1 × 10^9^ CFU/ml Rev. 1, or 1 × 10^9^ CFU/ml B. melitensis 16M. Control groups received 1 ml of PBS via subcutaneous injection. Animals were monitored daily for adverse events or until parturition occurred.

### Bacterial colonization of ewes.

**FIG 2:**
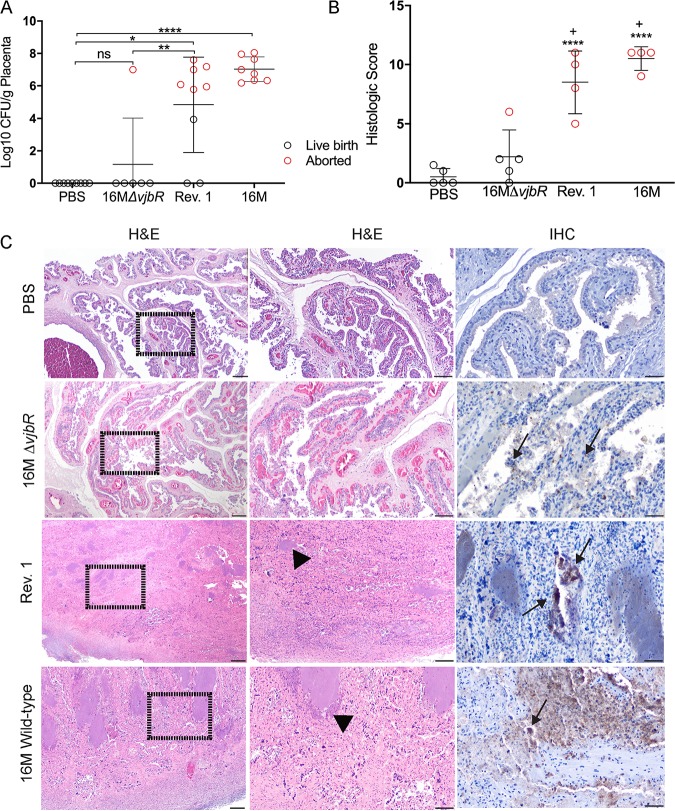
Bacteriological, histopathological, and immunohistochemical evaluation of placenta. (A) The placenta was collected at the time of abortion or parturition and cultured on Farrell’s media to assess colonization. The horizontal bar indicates the mean. *P* values were determined by ANOVA followed by Tukey’s multiple comparisons. Values that are significantly different are indicated by bars and asterisks (*, *P < *0.05; **, *P < *0.01; ****, *P < *0.0001). The only ewe with placental colonization in the 16M*ΔvjbrR* group aborted on day 110 of gestation (40 days postvaccination). (B) The placenta was graded in a blind fashion for edema (0 to 1), mononuclear infiltrate (0 to 4), fibrosis (0 to 4), necrosis (0 to 4), and bacteria (0 to 1). Differences were compared between PBS and vaccinated groups (+, *P* < 0.05) or 16M*ΔvjbrR* and Rev. 1 and 16M (****, *P* < 0.0001). (C) Representative sections of hematoxylin and eosin (H&E)-stained and immunohistochemical (IHC)-labeled placenta at the time of abortion or parturition. In the ewe from the 16M*ΔvjbrR* group that aborted, the placenta had mild lesions of inflammation and necrosis (arrows) with intracellular *Brucella* antigen. Placenta samples from Rev. 1-and 16M B. melitensis-vaccinated pregnant sheep that aborted had a severe necrotizing placentitis (middle column, arrowheads) with abundant intracellular and extracellular *Brucella* antigen (right column, arrows). H&E left column, 10×; bar = 100 μm. H&E middle column, 10×; bar = 100 μm. IHC 1:2,000 anti-*Brucella* antibody right column, 40×; bar = 50 μm.

To determine if failure to abort was due to decreased placental colonization, the placenta was collected at the time of abortion or parturition for microbiological culture on Farrell’s media. No growth was demonstrated in placenta from ewes administered sterile PBS or in 5 of 6 (83.3%) of 16M*ΔvjbR* ewes; in contrast, Rev. 1 and 16M had a mean colonization of between 10^6^ to 10^8^ CFU/g, respectively (Fig. 2A). Placental colonization was noted in 1 of 6 (16.7%) ewes vaccinated with 16M*ΔvjbrR*, which occurred in conjunction with a spontaneous abortion at 40 days postvaccination. 16M*ΔvjbR* had a statistically lower level of placental colonization than Rev. 1 (*P < *0.01), and thus, failure to colonize the placenta is a feature of vaccine safety.

Placentae were then evaluated by light microscopy for pathological changes associated with placental colonization. Specifically, the placenta was graded for evidence of an inflammatory response, including edema, inflammatory cell infiltrate, necrosis, and presence of bacteria (see Table S1 in the supplemental material). Placentas from all 6 PBS controls and 5 of 6 16M*ΔvjbrR* ewes had no significant pathology and an average group histologic score of 0 and 3, respectively (Fig. 2B). The placenta from the 16M*ΔvjbrR* ewe that aborted had mild placentitis (Fig. 2C). In contrast, the average histologic score was 8 in Rev. 1 and 10.5 in 16M ewes (Fig. 2B), which correlated with severe necrotizing and neutrophilic placentitis (Fig. 2C). We further confirmed that the inflammatory response was due to *Brucella* infection by performing immunohistochemistry with a polyclonal anti-*Brucella* antibody, which demonstrated *Brucella* antigen within inflammatory foci (Fig. 2C).

10.1128/mSphere.00120-20.1 TABLE S1 Histologic criteria for evaluating inflammation of the placenta. Download Table S1, DOCX file, 0.01 MB. Copyright © 2020 Hensel et al. 2020 Hensel et al. This content is distributed under the terms of the Creative Commons Attribution 4.0 International license.

Infected placentas from abortion events are an important means of spreading the disease within a flock, and thus, a vaccine that fails to colonize placenta or induce abortions is expected to reduce shedding and environmental contamination with a resultant reduced threat to other animals and humans (8). A B. melitensis mutant, BM*virB2*, which was attenuated in pregnant goats, had less inflammation in tissues examined by light microscopy (17). Similarly, a combination of minimal colonization and tissue pathology in the 16M*ΔvjbrR* group in pregnant sheep indicates that the attenuation reduced placental colonization and could offer a safer LAV for use in pregnant animals with potential translation into a vaccine candidate for humans.

### Fetal colonization.

Vertical transmission occurs when an infection spreads from the dam to the fetus and is another factor to consider when evaluating vaccine safety. B. melitensis can be vertically transmitted and potentially lead to latent carriers in replacement ewe lambs, which could perpetuate disease in a flock, as congenitally/latently infected lambs may experience abortions at the first breeding (18–20). Vertical transmission was evaluated in an experimental infection of pregnant goats with wild-type Brucella melitensis and Rev. 1, and 92% and 43% of the offspring, respectively, had recoverable organisms from liver, spleen, lung, abomasum, and/or abomasal contents. A similar rate of transmission could be expected in the pregnant sheep model (14).

**FIG 3:**
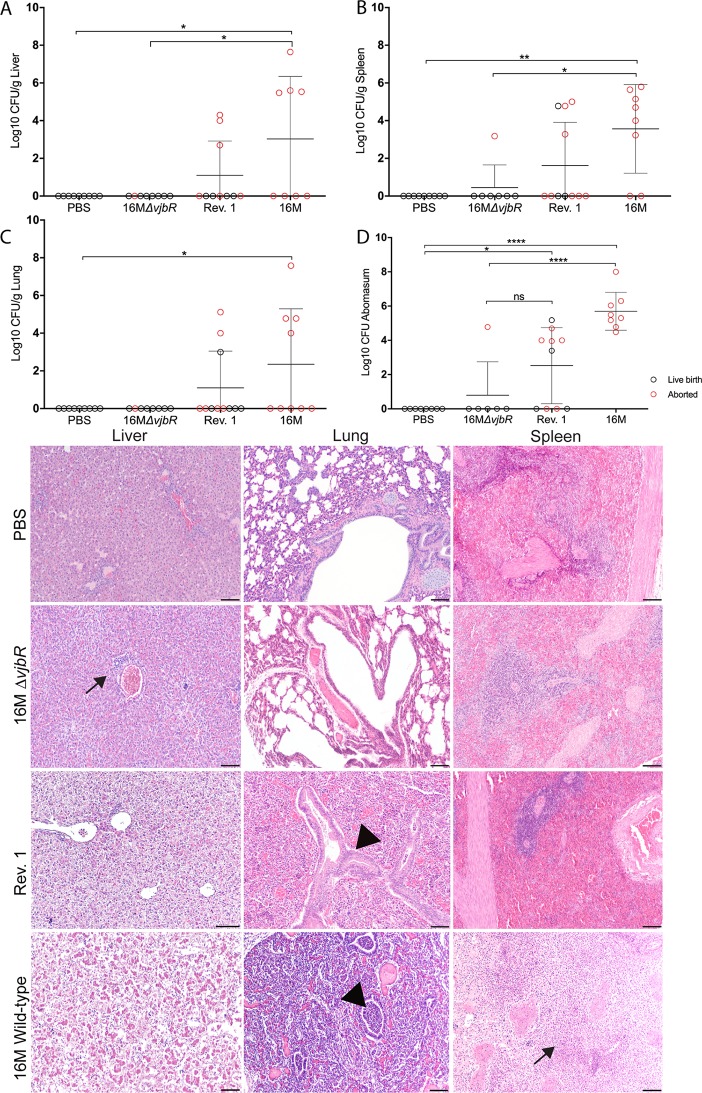
Vertical transmission of *Brucella* sp. from dam to offspring. From each group of 6 ewes, pregnancy resulted in various numbers of offspring, namely, 16M*ΔvjbrR*, *n* = 7; Rev. 1, *n* = 11; 16M, *n* = 9; and PBS, *n* = 9. In some cases, tissues could not be collected from aborted fetuses. Fetal liver (A), spleen (B), lung (C), and abomasal contents (D) were cultured on Farrell’s media to evaluate if vaccination or infection of the ewe resulted in vertical transmission of the vaccine strain to the fetus/offspring. The horizontal bar indicates the mean. *P* values were determined by 2-way ANOVA followed by Tukey’s multiple comparisons. Values that are significantly different are indicated by bars and asterisks (*, *P < *0.05, **, *P < *0.01, ****, *P < *0.0001). (E) Representative H&E images of spleen, liver, and lung at ×10 magnification. No significant histopathological changes were seen in the tissues from the PBS or 16M*ΔvjbrR* groups, which had live births. A moderate neutrophilic bronchopneumonia consistent with neonatal brucellosis was noted in the lungs of two aborted lambs from the Rev. 1 and 16M B. melitensis groups (arrowheads). The aborted fetus from the 16M*ΔvjbrR* group had a minimal periportal infiltrate of lymphocytes (arrow). An aborted fetus from the 16M B. melitensis group had areas of necrosis in the spleen (arrow). H&E bar = 100 μm.

Targets of fetal infection, which included spleen, liver, lung, and abomasal contents, were assessed to characterize the extent of vertical transmission. Similar to adult animals, *Brucella* spp. have a tropism for fetal reticuloendothelial organs, but abomasal contents are also important to culture because pathogens that cross the placenta will be in the amniotic fluid ingested by fetuses while *in utero* (21). Vertical transmission was evident in at least one target organ in 9 of 9 (100%) aborted fetuses from 16M B. melitensis-infected ewes, which further indicates that we have correctly modeled events that occur during natural infection. Colonization is not uniformly distributed in the offspring; however, the abomasal contents appear to be the tissue target that gives the best opportunity to detect vertical transmission because 9 of 9 (100%) 16M, 6 of 11 (54.5%) Rev. 1, and 1 of 7 (14.2%) 16M*ΔvjbrR* offspring were culture positive. No significant differences were noted in the mean CFU/g recovered from the spleen, liver, lung, and abomasal contents of the Rev. 1 and 16M*ΔvjbrR* offspring (Fig. 3). While not statistically significant, 16M*ΔvjbrR* seems less likely to result in vertical transmission because the only fetus from 16M*ΔvjbrR* with colonization detected in the spleen and abomasal contents was aborted, whereas both aborted fetuses and live births from the Rev. 1-vaccinated ewes had evidence of vertical transmission (Fig. 3A to D).

Colonization results were corroborated by evaluating spleen, liver, and lung via histopathology for an inflammatory response in the offspring. When evaluating histopathologic changes in aborted fetuses, it is important to distinguish between the effects of autolysis or tissue decomposition from inflammation due to a disease process. Offspring from the PBS and 16M*ΔvjbrR* groups had no significant pathology in any of the tissues examined. The most dramatic lesions were seen in aborted 16M fetuses, which had splenic necrosis and neutrophilic inflammation (3 of 9) and 2 of 9 had neutrophilic bronchopneumonia (Fig. 3E). Within the Rev. 1 cohort, 2 of 11 fetuses had a similar neutrophilic bronchopneumonia (Fig. 3E). Neutrophilic pneumonia, including both interstitial and bronchopneumonia patterns, are common lesions in ruminants infected with *Brucella* spp. and can occur independently of abortion (22, 23).

### Humoral response to vaccination.

**FIG 4:**
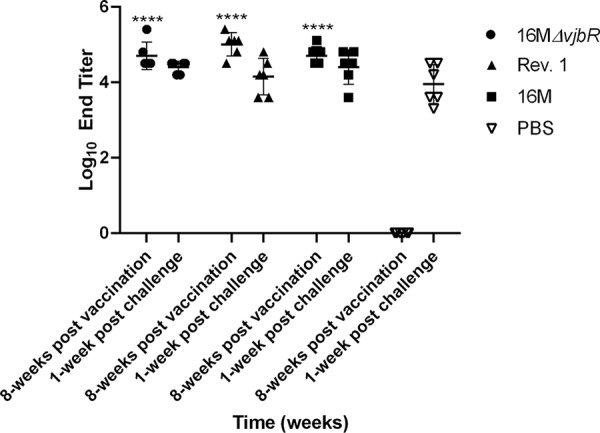
Evaluation of the *Brucella* sp.-specific IgG response to vaccination with PBS, 16M*ΔvjbrR*, Rev. 1, or 16M B. melitensis. Data represent log_10_ of the end titers per group (*n* = 6) at 8 weeks postvaccination, which corresponds to the time period of abortions and 1-week postchallenge. The difference between time points and groups was determined by 2-way ANOVA followed by Tukey’s multiple comparisons. Values that are significantly different from PBS controls are indicated by asterisks. (****, *P < *0.0001).

Infection with wild-type *Brucella* spp. and vaccination with LAV both elicit a *Brucella*-specific humoral immune response (15, 16, 24). Previous studies have shown that the *Brucella*-specific IgG levels can be used to determine the serological response to vaccination in the mouse and goat model (10–12, 17, 25). The IgG enzyme-linked immunosorbent assay (ELISA) utilized in this study was previously validated in goats and was performed using serum samples collected every 2 weeks to evaluate the humoral response to vaccination and its duration (17). Because the ELISA coating antigen is a heat-killed extract of a smooth strain, it can be used to detect the serological response to smooth variants, including vaccines strains, such as Rev. 1 and 16M*ΔvjbR*, as well as the response to virulent organisms. The PBS-vaccinated group did not seroconvert until challenged with B. melitensis (Fig. 4). A *Brucella*-specific antibody response developed 2 to 3 weeks postvaccination with 1 × 10^9^ CFU/ml 16M, 1 × 10^9^ CFU/ml Rev. 1, and 1 × 10^10^ CFU/ml 16M*ΔvjbR* (Fig. S1) and was statistically increased 6 weeks postvaccination in the Rev. 1 (*P < *0.05) and 16M (*P < *0.01) groups compared with the naive controls during the time period of abortions (Fig. 4). Overall, the level of anti-*Brucella*-specific IgG at each time point correlated with virulence of the vaccine; thus, the *Brucella*-specific IgG response was highest in the 16M wild type, intermediate in Rev. 1, and lowest in 16M*ΔvjbR*. The antibody response in ewes inoculated with Rev. 1 and 16M persisted throughout the 16 weeks of the study (S1), which was anticipated based on previous research in experimental and natural models of infection that have demonstrated antibody responses for up to 151 weeks postvaccination or infection (7, 20, 26). The end titer was not statistically significant 1 weeks postchallenge with B. melitensis, indicating that the immune response was similar between groups (Fig. 4). The duration of immunity for 16M*ΔvjbR* has not been fully established in the natural host; however, in the mouse model, *ΔvjbR* was protective 20 weeks postvaccination (10). We can conclude from this study that the *Brucella*-specific IgG response to vaccination with 16M*ΔvjbR* persists for at least 16 weeks.

10.1128/mSphere.00120-20.2 FIG S1 Evaluation of the *Brucella* sp.-specific IgG response to vaccination with PBS, 16M*ΔvjbrR*, Rev. 1, or 16M B. melitensis. Data represent the mean absorbance per group per time point. Statistical significance was determined by 2-way ANOVA followed by Tukey’s multiple comparisons. Values that are significantly different are indicated by asterisks. (* *P < *0.05, ** *P < *0.01, *** *P < *0.001, **** *P < *0.0001). Download FIG S1, TIF file, 0.05 MB. Copyright © 2020 Hensel et al. 2020 Hensel et al. This content is distributed under the terms of the Creative Commons Attribution 4.0 International license.

### Efficacy in postpartum sheep.

**FIG 5:**
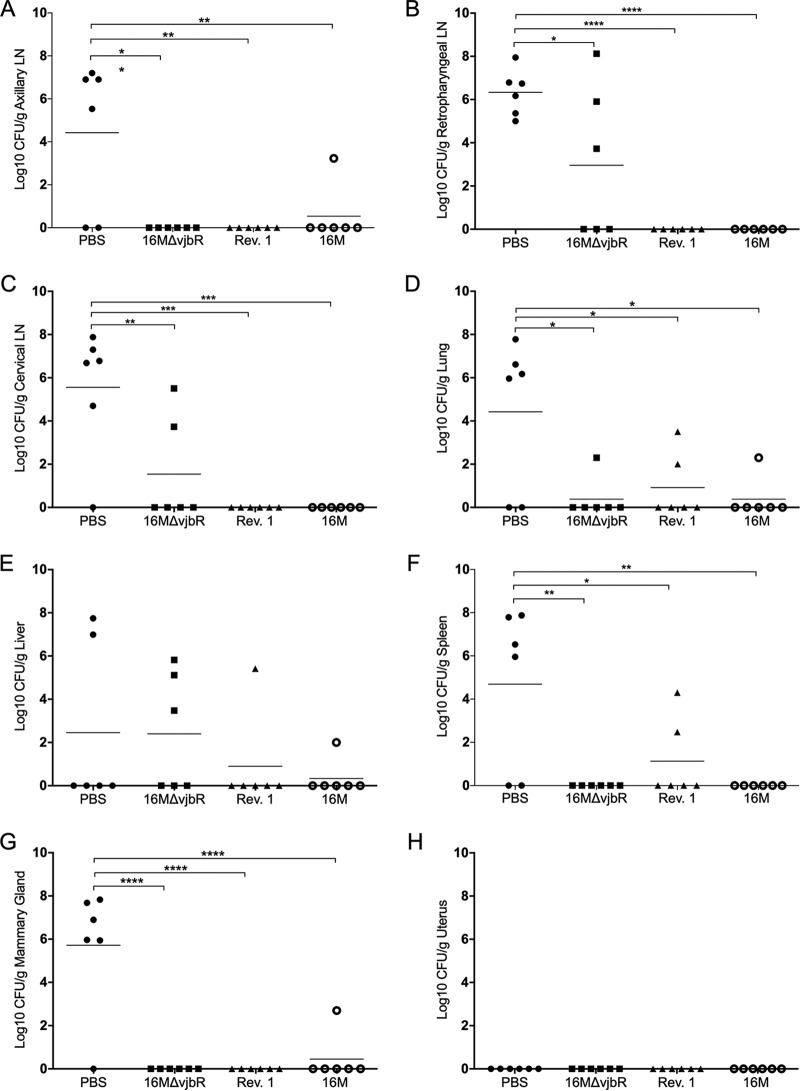
Evaluation of vaccine efficacy in postpartum ewes previously vaccinated with PBS, 16M*ΔvjbrR*, Rev. 1, or 16M B. melitensis. Bacterial burden 1 week postchallenge in the axillary lymph node (A), retropharyngeal lymph node (B), cervical lymph node (C), lung (D), liver (E), spleen (F), mammary gland (G), and uterus (H). The horizontal bar indicates the mean. *P* values were determined by ANOVA followed by Tukey’s multiple comparisons. Values that are significantly different are indicated by bars and asterisks (*, *P < *0.05, **, *P < *0.01, ***, *P < *0.001, ****, *P < *0.0001).

The ability of the vaccine to reduce systemic infection was assessed by challenging all ewes at 5 weeks postpartum (17 weeks postvaccination) with 1 × 10^7^ CFU/ml B. melitensis 16M via bilateral conjunctival inoculation. Efficacy was determined by enumerating bacterial colonization of target organs, such as the reticuloendothelial system (spleen, liver, axillary lymph node, retropharyngeal lymph node, cervical lymph node, and lung) and reproductive tissues (uterus and mammary gland). One week after challenge, 6 of 6 (100%) naive animals had recoverable bacteria from at least one target organ, indicating the challenge dose could induce disease (Fig. 5). As expected, 5 of 6 (83.3%) nonpregnant ewes previously challenged with 16M did not have recoverable organisms from the tissues evaluated because the primary infection provided protection against a secondary infection (7, 27, 28). It is well documented that an initial infection with a pathogen can provide protection against a secondary infection with the same or similar pathogens due to a convergence of acquired immune factors that stimulate a rapid response to infection (27, 28). Even though 16M*ΔvjbR* is attenuated compared with Rev. 1 and 16M, it appeared to limit colonization after challenge. Additional studies will be required to evaluate the efficacy of 16M*ΔvjbR* to determine if protection is similar to that offered by Rev. 1.

Both 16M and Rev. 1 have the potential for excretion in the milk, which not only could be a source of bacteria for lambs but also, if not properly pasteurized, could pose a risk for human infection (14, 29, 30). The mean colonization of the mammary gland in the naive group was statistically increased (*P < *0.0001) compared with both vaccine strains and 16M (Fig. 5G). 16M colonizes the mammary gland and could be shed in the milk. Previous infection with 16M or vaccination with Rev. 1 or 16M*ΔvjbR* appeared efficacious and prevented reinfection in postpartum ewes challenged with the wild type.

**FIG 6:**
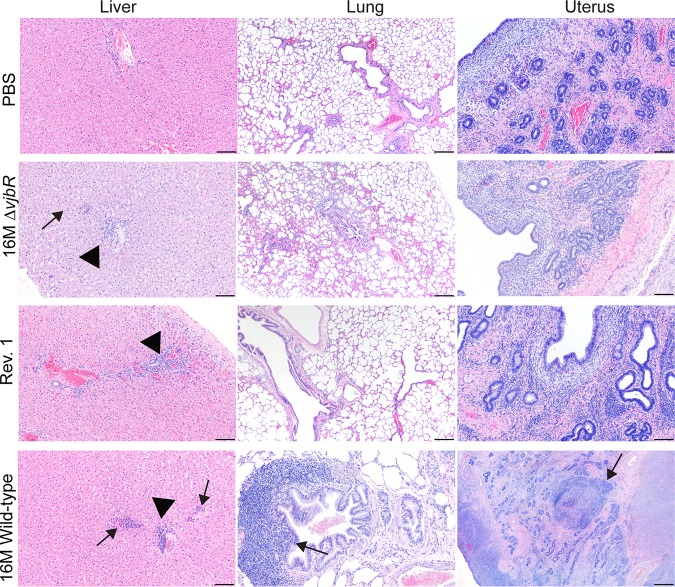
Representative H&E-stained section of liver (left column), lung (middle column), and uterus (right column) of postpartum sheep from each group (PBS, 16M*ΔvjbrR*, Rev. 1, 16M wild type) 1 week postchallenge with 1 × 10^7^ CFU/ml B. melitensis 16M. No microscopic lesions were observed in any tissue in the naive/PBS group. In contrast, sheep previously vaccinated with 16M*ΔvjbrR*, Rev. 1, or 16M had multifocal random foci of neutrophilic and histiocytic inflammation in the liver parenchyma (arrows) and periportal infiltrates of lymphocytes and plasma cells (arrowheads). A single ewe in the 16M group had proliferation of the bronchiole associated lymphoid tissue (arrow). No lesions were observed in the uterus of 16*ΔvjbrR* or Rev. 1 groups; a single ewe in the 16M group had multifocal areas of necrosis surrounded by an intense inflammatory reaction in the endometrial stroma (arrow), which was attributed to the previous infection with 16M, as no bacteria were recovered from culture. Hematoxylin and eosin (H&E), 10×; bar = 100 μm.

Tissue pathology was also evaluated following challenge with 16M B. melitensis to determine if vaccination prevented or reduced the inflammatory response to infection. No significant lesions were noted in the naive (PBS) group following challenge in any tissue despite the presence of recoverable bacteria, which is likely due to the short duration of the study. In small animal experimental models of infection with *Brucella* spp., colonization is noted 1 week postchallenge, but the inflammatory response often lags and is developed in the majority of animals by 14 days postchallenge (31, 32). The most significant histologic findings in ewes from 16M*ΔvjbR* (3 of 6), Rev. 1 (1 of 6), and 16M (1 of 6) groups were random foci of neutrophilic inflammation and lymphoplasmacytic periportal infiltrates in the liver. In the 16M and Rev. 1 groups, no brucellae were cultured postchallenge, and so the liver lesions in these two sheep represent persistent and unresolved inflammation from the primary infection (Fig. 6). However, in the 16M*ΔvjbR* group, inflammation coincided with colonization of the tissue. Hepatic inflammation often develops in humans with brucellosis and has been noted in both large animal and lab animal experimental models (17, 31, 33). Typical light microscopic findings include neutrophilic hepatitis and lymphoplasmacytic periportal hepatitis (33).

An interesting question to investigate was whether vaccination protected against colonization of the nongravid (pregnant) uterus. If *Brucella* sp. colonizes the nongravid uterus, then the bacteria could await the preferential conditions that occur during pregnancy to replicate and cause inflammation. The uterus was not colonized and had no significant inflammation in the majority of ewes from each group (Fig. 6). In 1 of 6 (16.7%) ewes previously infected with 16M, the uterine endometrium had multifocal areas of necrosis surrounded by large numbers of neutrophils and macrophages, which indicates that inflammation from the primary infection persists for up to 16 weeks postinfection.

### Conclusions.

This study successfully modeled the events which occur during natural infection and surveyed all of the appropriate tissue targets to evaluate safety and protection through a combination of bacteriological culture and histopathology. This holistic approach is important to evaluate the ability of a vaccine candidate to limit or prevent colonization as well as to protect against an inflammatory response in target organs. This study demonstrates the importance of evaluating vaccine candidates in natural host models because 16M*ΔvjbR* provided sterile immunity combined with minimal tissue pathology in the mouse model, but information gleaned from these studies did not predict the retained virulence demonstrated in pregnant sheep (10–12).

The goal of vaccination with LAV is to decrease clinical signs and shedding rather than provide sterile immunity; therefore, 16M*ΔvjbR* should be considered an improvement upon Rev. 1 due to decreased abortions and limited colonization and inflammation of tissue targets. By limiting the abortion potential, 16M*ΔvjbR* reduces environmental contamination, which protects both animal and human health. However, the attenuation level of 16M*ΔvjbR* may not be enough to completely reduce the risk of abortion, especially if the vaccine is given during the most susceptible stage of gestation or at extremely high doses. Dose titration or adding a second mutation could increase the attenuation of 16M*ΔvjbR* and further minimize adverse side effects of vaccinating pregnant animals. In conclusion, 16M*ΔvjbR* is a promising vaccine candidate that should be further evaluated to refine the dose and evaluate efficacy in pregnant sheep.

## MATERIALS AND METHODS

### Bacterial strains and cultures.

B. melitensis biovar 1 strain 16M was originally acquired from the lung of an aborted goat (34). Commercially available Rev. 1 vaccine OviRev (Vetoquinol) was prepared from frozen suspension, harvested, and diluted to approximately 1 × 10^9^ CFU/ml using a Klett meter. B. melitensis 16MΔ*vjbR* was derived from our laboratory stock. All wild-type and vaccine strains were grown on tryptic soy agar (TSA) or tryptic soy broth (TSB) at 37°C in an atmosphere containing 5% (vol/vol) CO_2_ for 72 h. Immunization and inoculum doses were verified by retrospective serial dilution, plating, and enumeration of colonies.

### Animals.

Twenty-four, 2-year-old mature cross-bred female sheep were acquired from a privately owned flock and upon arrival at Colorado State University facilities were tested for specific anti-*Brucella* immunoglobulin G (IgG) levels by enzyme-linked immunosorbent assay (ELISA). Sheep were housed in an outdoor, restricted access, large-animal isolation facility operated under guidelines approved by the United States Department of Agriculture/Animal and Plant Health Inspection Service (USDA/APHIS). Ewes (*n* = 24) were synchronized into estrus and impregnated via natural breeding. All rams were negative for Brucella melitensis and Brucella ovis. All experimental procedures and animal care were performed in compliance with institutional animal care regulations.

### Ethics statement.

This study was carried out in accordance with Animal Welfare Act regulations by the United States Department of Agriculture (USDA) and Public Health Service Policy on Humane Care and Use of Laboratory Animals (PHS Policy) administered by the National Institutes of Health (NIH). All animal research was conducted under a protocol approved by Texas A&M University and Colorado State University Institutional Animal Care and Use Committees (IACUC), in an Association for Assessment and Accreditation of Laboratory Animal Care (AAALAC)-accredited animal facility. Animal welfare was monitored on a daily basis, and all efforts were made to minimize suffering.

### Immunization.

At 2 months postbreeding, the ewes were evaluated by ultrasonography to confirm pregnancy and were then moved to an agriculture biosafety level 3 (ABSL3) facility at Colorado State University for the duration of the experiment. Animals were acclimated to the ABSL3 space for 10 days prior to immunization. At approximately 70 days of gestation, animals were randomly assigned to 1 of 4 groups (*n* = 6), which were housed separately by group under similar conditions. Each pregnant sheep received a single subcutaneous vaccine dose of either 1 × 10^10^ CFUs 16M*ΔvjbR*, 1 × 10^9^ CFU/ml Rev. 1, 1 × 10^9^ CFU/ml B. melitensis 16M, or 1 ml of sterile PBS.

### Safety study in pregnant sheep.

Pregnant sheep (ewes) were implanted with a subcutaneous microchip (LifeChip, Destrong Industries) in the right axilla to monitor body temperature once daily throughout the experiment using a handheld DAS-7000 reader (BioMedic Data Systems). Ewes were monitored twice daily for abortions. Aborted fetuses were immediately collected, and necropsy was performed to collect samples for culture and histology. The placenta was collected at the time of abortion; samples were vigorously rinsed with sterile PBS, and then 100 mg of tissue was homogenized in 0.9 ml of sterile PBS (Gibco) using a TissueLyser II (Qiagen) for 5 min at 25 cycles/min. After serial dilutions, 100 μl of each dilution was plated on Farrell’s media and incubated at 37°C in an atmosphere containing 5% (vol/vol) CO_2_ for 72 h, and colonies were counted (17).

To evaluate vertical transmission of infection from the ewe to the fetus, 100 mg of fetal lung, liver, spleen, and abomasal fluid were collected for bacterial culture as described above. Finally, the aforementioned tissues were collected and fixed in 10% neutral buffered formalin for histopathology. In the case of uneventful delivery, lambs were euthanized immediately after birth via intravenous overdose of sodium pentobarbital and the above-described tissues were collected for culture and histology.

### Efficacy study in postpartum ewes.

To assess vaccine efficacy in nonpregnant sheep, all ewes were challenged 5 weeks after the PBS group gave birth with 1 × 10^7^ CFU B. melitensis 16M via conjunctival inoculation. At 1 week postchallenge, ewes were euthanized by intravenous overdose of sodium pentobarbital and necropsied, and samples of uterus, spleen, liver, lung, mammary gland, axillary lymph node, cervical lymph node, and retropharyngeal lymph node were collected for bacteriology as described above.

### Histopathology.

Tissues from the ewes and fetuses were fixed in 10% neutral buffered formalin (NBF) for a minimum of 48 h. Tissues were routinely processed and embedded, sectioned at 5 μm, and stained with hematoxylin and eosin. A grading scheme for evaluating placental inflammation based on edema (0 to 1), mononuclear infiltrate (0 to 4), fibrosis (0 to 4), necrosis (0 to 4), and bacteria (0 to 1) was developed and applied to sections of placenta in a blind fashion by a board-certified veterinary anatomic pathologist (Table S1).

### Immunohistochemistry.

Five micrometer sections of placenta were adhered to positively charged glass slides (Mercedes Medical). Following deparaffinization and rehydration through a series of xylene and ethanol steps, antigen retrieval and blocking were performed as previously described (35). Briefly, the antigen was unmasked using a 1× solution of electron microscopy buffer A (Electron Microscopy Services, Hatfield, PA) in a 2100 antigen retriever (Aptum Biologics Ltd.), according to the manufacturer’s protocol. Primary incubation with a 1:2,000 dilution of a *Brucella* polyclonal rabbit antibody (Bioss) was performed in a humidifying chamber overnight at 4°C. Negative-control tissues were incubated with rabbit nonimmune serum diluted in PBS. A biotinylated secondary anti-rabbit antibody (Vectastain ABC) was used following primary incubation, and the antigen was visualized using Betazoid DAB chromogen kits (Biocare Medical) according to the manufacturer’s instructions. The slides were counterstained with Meyer’s hematoxylin III and cover slipped.

### Serology.

Approximately 10 ml of blood was collected from the jugular into serum separator tubes at 2-week intervals for throughout the study (16 weeks). Blood was allowed to clot overnight and was then centrifuged at 1,700 × *g* for 25 minutes at room temperature. A previously validated anti-*Brucella*-specific immunoglobulin G (IgG) indirect enzyme linked immunosorbent assay (iELISA) was used as previously described (17). Briefly, a 96-well plate was coated with 25 ng/well of Brucella abortus 2308 heat-killed lysate and held overnight at 4°C. Plates were washed three times with phosphate-buffered solution plus 0.5% Tween 20 (PBS-T) and were blocked with 0.25% bovine serum albumin (Sigma) for 2 h at room temperature. Sheep serum samples were diluted in blocking buffer (0.25% [wt/vol] bovine serum albumin) to 1:2,000, and 100 μl was added to the plates and incubated at 37°C for 1 h. Plates were washed five times with PBS-T, and then peroxidase-labeled anti-sheep secondary IgG antibody was added at 1:1000, followed by incubation at 37°C for 1 h. After a final washing step, horseradish peroxidase substrate (Sigma) was added, and plates were incubated for 30 minutes at 37°C. Absorbance was measured at 450 nm. All assays were performed in triplicate, and the results are presented as the mean value per group plus standard deviation.

### Statistical analysis.

The analysis was performed using GraphPad Prism 6.0 software. Statistical analysis of ELISA and CFU data was performed using two-way analysis of variance (ANOVA) followed by Tukey’s multiple-comparison test. Histopathologic scores were compared using the Mann-Whitney U test.
