# Supplementary material for: Vaccine Candidate Brucella melitensis 16MΔvjbR Is Safe in a Pregnant Sheep Model and Confers Protection
Source: mSphere. 2020 May 13;5(3):e00120-20. doi: 10.1128/mSphere.00120-20 (PMC7227765; doi:10.1128/mSphere.00120-20)
Supplement: TABLE S1 [file mSphere.00120-20-st001.docx]

Table S1. Histologic criteria for evaluating inflammation of the placenta.

| Placenta | Score | Description |
| --- | --- | --- |
| Edema  Mononuclear infiltrate | 0  1  0 | Not present  Present  Not present |
|  | 1 | Minimal—One focus per 5 10x objective |
|  | 2 | Mild—Two to four foci per 5 10x objective |
|  | 3 | Moderate—Five to 10 foci per 5 10x objective |
|  | 4 | Marked—>8 per 5 10x objective |
| Fibrosis | 0 | Not present |
|  | 1 | Minimal—One focus per 5 10x objective |
|  | 2 | Mild—Two to four foci per 5 10x objective |
|  | 3 | Moderate—Five to 7 foci per 5 10x objective |
|  | 4 | Marked—>8 per 4x objective |
| Necrosis | 0 | Not present |
|  | 1 | Minimal—one focus per 5 10x objective |
|  | 2 | Mild—Two to four foci per 5 10x objective |
|  | 3 | Moderate—Five to 7 foci per 5 10x objective |
|  | 4 | Marked— >8 foci per 5 10x objective |
| Bacteria | 0  1 | Not present  Present |
